# Characterization of a novel microfilarial antigen for diagnosis of *Wuchereria bancrofti* infections

**DOI:** 10.1371/journal.pntd.0010407

**Published:** 2022-05-23

**Authors:** Sarah E. Greene, Kerstin Fischer, Young-Jun Choi, Kurt C. Curtis, Philip J. Budge, Makedonka Mitreva, Christopher L. King, Peter U. Fischer, Gary J. Weil

**Affiliations:** 1 Infectious Diseases Division, Department of Pediatrics, Washington University School of Medicine, St Louis, Missouri, United States of America; 2 Infectious Diseases Division, Washington University School of Medicine, St Louis, Missouri, United States of America; 3 McDonnell Genome Institute, Washington University School of Medicine, St. Louis, Missouri, United States of America; 4 Department of Genetics, Washington University School of Medicine, St. Louis, Missouri, United States of America; 5 Center for Global Health and Diseases, Department of Pathology, Case Western Reserve University and Veterans Affairs Research Service, Cleveland, Ohio, United States of America; Stanford University, UNITED STATES

## Abstract

**Background:**

Lymphatic filariasis (LF) is a neglected tropical disease caused by the filarial nematodes *Wuchereria bancrofti*, *Brugia malayi and Brugia timori*. The Global Program to Eliminate LF uses mass drug administration (MDA) of anti-filarial drugs that clear microfilariae (Mf) from blood to interrupt transmission by mosquitos. New diagnostic tools are needed to assess the impact of MDA on bancroftian filariasis, because available serologic tests can remain positive after successful treatment.

**Methodology/Principal findings:**

We identified *Wb-bhp-1*, which encodes a *W*. *bancrofti* homologue of BmR1, the *B*. *malayi* protein used in the Brugia Rapid antibody test for brugian filariasis. *Wb-bhp-1* has a single exon that encodes a 16.3 kD protein (Wb-Bhp-1) with 45% amino acid identity to BmR1. Immunohistology shows that anti-Wb-Bhp-1 antibodies primarily bind to Mf. Plasma from 124 of 224 (55%) microfilaremic individuals had IgG4 antibodies to Wb-Bhp-1 by ELISA. Serologic reactivity to Wb-Bhp-1 varied widely with samples from different regions (sensitivity range 32–92%), with 77% sensitivity for 116 samples collected from microfilaremic individuals outside of sub-Saharan Africa. This variable sensitivity highlights the importance of validating new diagnostic tests for parasitic diseases with samples from different geographical regions. Individuals with higher Mf counts were more likely to have anti-Wb-Bhp-1 antibodies. Cross-reactivity was observed with a minority of plasma samples from people with onchocerciasis (17%) or loiasis (10%). We also identified, cloned and characterized BmR1 homologues from *O*. *volvulus* and *L*. *loa* that have 41% and 38% identity to BmR1, respectively. However, antibody assays with these antigens were not sensitive for onchocerciasis or loiasis.

**Conclusions:**

Wb-Bhp-1 is a novel antigen that is useful for serologic diagnosis of bancroftian filariasis. Additional studies are needed to assess the value of this antigen for monitoring the success of filariasis elimination programs.

## Author summary

Lymphatic filariasis (LF) is a highly disabling and stigmatizing disease caused by parasitic worms that are transmitted by mosquitoes. There is a coordinated global effort to eliminate LF based on mass drug administration (MDA) of donated anti-filarial medications. Improved methods are needed to determine when transmission of the infection has been interrupted in previously endemic areas so that MDA can be safely stopped. This paper reports the discovery and characterization of a novel *W*. *bancrofti* antigen, Wb-Bhp-1, which is a homologue of the *Brugia malayi* protein used in antibody tests to monitor filariasis elimination in areas of Asia where LF is caused by *Brugia* species. We show that a test for IgG4 antibodies to Wb-Bhp-1 was fairly specific for *W*. *bancrofti* infection. However, the sensitivity of this test varied by the geographic origin of the samples. Sensitivity was highest for samples collected in the Indo-Pacific region and lowest for samples collected in Côte d’Ivoire. Geographic differences in the parasite or the human immune responses to infection may account for this variability. This range in sensitivity highlights the importance of validating new diagnostic tests for parasitic diseases with samples from different geographical regions.

## Introduction

Lymphatic filariasis (LF) is a deforming and disabling disease caused by parasitic nematodes that are transmitted by mosquitoes. Since its inception in 2000, the Global Programme to Eliminate LF has made impressive progress by reducing the estimated numbers of infected people from 120 million to 51 million [1]. This accomplishment demonstrates the effectiveness of the mass drug administration (MDA) strategy, which involves distributing anti-filarial medications to kill the larval stage of the parasite (microfilariae, Mf). The combination of ivermectin, diethylcarbamazine (DEC) and albendazole (IDA) is currently the most effective MDA regimen for clearing Mf, but results in slow clearance of filarial antigenemia [2,3]. The drug combinations used for MDA depend on whether there are other co-endemic parasitic infections in an area. Improved diagnostic tests are needed to better identify areas where MDA has been successful enough that it can be halted. Indeed, the World Health Organization (WHO) lists the development of improved diagnostics as an important priority in their 2020 Roadmap for LF elimination by 2030 [4].

Current guidelines call for transmission assessment surveys (TAS) after five or more rounds of MDA to determine whether MDA can be halted in a given area. TAS rely on detection of circulating filarial antigen (CFA) in the blood with tests such as the Filariasis Test Strip (FTS) or, in areas with brugian filariasis, on detection of anti-filarial antibodies with the Brugia Rapid test [5]. CFA tests are useful for mapping endemic areas and for assessing the impact of MDA on filariasis transmission in sentinel groups. However, TAS surveys have been shown to be insensitive tools for this purpose in some settings [6–8]. Furthermore, CFA tests in adults are a lagging indicator of MDA success, because CFA often remains detectable in human blood for years after treatment has cleared Mf [3,9].

Antibodies to some filarial antigens become detectable in blood sooner after exposure or infection than Mf or CFA [10]. Furthermore, antibody assays may be more sensitive than tests for CFA or Mf for detecting filarial infections or exposure to infection in children [9,11]. Usually, school-aged children are sampled during TAS. Therefore, antibody tests might be useful to detect whether children have been exposed to filarial parasites. Similarly, an antibody test that correlates well with the presence of microfilaremia could be useful for use in post-MDA population surveys. There are several available antibody tests for LF; the most commonly used commercially available tests detect antibodies to the filarial antigens Bm14, BmR1 or Wb123 [12–16]. The Brugia Rapid test, which is used in *Brugia* endemic areas, detects IgG4 antibody to the *Brugia malayi* protein BmR1 [16]. The function of BmR1 is unknown, but it is expressed in Mf and female worms [17,18]. The main drawback of the Bm14 or Wb123 antibody tests is that antibodies to those antigens remain detectable for years after clearance of the infection [13,15]. In contrast, antibodies to BmR1 often clear by 2 years after treatment, making anti-BmR1 antibody testing especially useful for MDA stopping decisions in areas with brugian filariasis [15,19]. The development of an antibody test for *W*. *bancrofti* that cleared within a few years after treatment could be very useful for MDA stopping decisions for bancroftian filariasis.

Based on the utility of the BmR1 antibody test, we hypothesized that antibody tests based on BmR1 homologues might be useful for diagnosis of other filarial parasites that infect humans, such as *W*. *bancrofti*, *Onchocerca volvulus*, and *Loa loa*. Approximately 40% of the global LF burden is in sub-Saharan Africa, where the latter two parasites are often co-endemic with *W*. *bancrofti* [20]. Indeed, some antibody tests for LF detect antibodies in serum samples from patients with onchocerciasis and/or loiasis that could lead to inaccurate assessments of LF endemicity in co-endemic areas, similar to what has been reported for CFA tests in Central Africa [21–23]. Thus, species specificity is especially important for diagnostic tests in areas of Africa with multiple co-endemic filarial infections.

Previously identified BmR1 homologues had 99–100% sequence identity to BmR1, and antibodies to these homologues did not prove to be sensitive or specific for bancroftian LF, onchocerciasis or loiasis [24]. This is a much higher degree of sequence identity than found in many other homologous genes in *W*. *bancrofti*, *B*. *malayi* and *O*. *volvulus*. Because there has been further sequencing of filarial parasite genomes since that publication, we hypothesized that additional BmR1 homologues might be present that have less sequence identity and that might be useful for sero-diagnosis. This paper reports the identification and partial characterization of these newly identified BmR1 homologues and early studies of their diagnostic potential.

## Methods

### BmR1 homologue identification and characterization

The BmR1 protein is encoded by the *B*. *malayi* gene *Bm17DIII* [16]. We identified a *W*. *bancrofti* BmR1 homologue, *Wb-bhp-1*, with a BLAST search for homologues of BmR1 on the WormbaseParasite database (Parasite.wormbase.org last accessed 3/18/21). We identified a second *W*. *bancrofti* BmR1 homologue, *Wb-bhp-2*, by an analysis of recently published *W*. *bancrofti* genomes [25]. BmR1 homologues from *O*. *volvulus* and *L*. *loa* were likewise identified using BLAST for homologues of BmR1 on WormbaseParasite. Amino acid alignment for these BmR1 homologues was conducted by ClustalV in MegAlign version 15 (DNAStar, Madison WI, USA). Percent identity was calculated for Wb-Bhp-1, Ov-Bhp-1 and Ll-Bhp-1 based on the amino acids conserved with BmR1 in the region of overlap.

### Genetic variation analysis

We analyzed previously published *W*. *bancrofti* genomic data from Haiti, Mali, Kenya, and PNG to assess sequence variation in Wb-Bhp-1 and Wb-Bhp-2 [25]. Sequencing reads were retrieved from the SRA (accession SRP056210 and SRP168632), adapter-trimmed using Trimmomatic v0.39 [26], and aligned to the *W*. *bancrofti* genome assembly (GenBank accession: GCA_005281725.1) using BWA-MEM v0.7.17 [27]. Polymerase chain reaction and optical duplicates were removed using Picard tools v2.22.0 and single-nucleotide variants were called via local de novo assembly of haplotypes using GATK v4.2.2 [28,29]. Variants were quality-filtered as previously described, and coding effects were predicted using SnpEff v5.0 [30,31]. VCFtools was used to summarize the variant allele frequencies in each population [32].

### Cloning and protein expression

All kits were used according to the manufacturer’s instructions. PCR amplification of each BmR1 homologous gene was conducted using blunt end PCR with Phusion DNA polymerase (New England Biolabs, Ipswich MA, USA), with an annealing temperature of 52°C and using the primers listed in S1 Table. *Wb-bhp-1* was amplified from *W*. *bancrofti* Mf genomic DNA obtained in Côte d’Ivoire from a person with *W*. *bancrofti* infection and no other filarial infection. It was possible to amplify this gene from genomic DNA as it had only 1 exon. *Wb-bhp-2* was amplified from double-stranded DNA synthesized by Integrated DNA Technologies based on available sequence for *Wb-bhp-2* in WormbaseParasite (IDT, Coralville IA, USA). *Ov-Bhp-1* was amplified from *O*. *volvulus* adult worm complementary DNA (cDNA) from Uganda. *Ll-Bhp-1* was amplified from double-stranded DNA synthesized by Integrated DNA Technologies based on available sequence for *Ll-bhp-1* in WormbaseParasite (IDT).

PCR products were visualized by agarose gel electrophoresis, and ligated into linearized pET100D plasmid, which incorporates an amino-terminal polyhistidine tag and an 8 amino acid Xpress tag (Invitrogen, Waltham MA, USA). The resultant vectors (S1 Table) were sequenced (Genewiz, South Plainfield NJ, USA), with sequence analysis on SeqManPro (DNAStar). Each plasmid was transformed into the BL21 *E*. *coli* strain for protein production. These strains were grown at 37°C in Luria broth with 50 ug/ml ampicillin to optical density at 600nm of 0.6, then induced with 1mM Isopropyl β-D-1-thiogalactopyranoside (IPTG) for 2 hours. Cells were collected by centrifugation at 4700rpm then subjected to a freeze/thaw cycle at -80°C. Cell pellets were then lysed with CelLyticB (Sigma, St Louis MO, USA) and benzonase. Protein was purified from the clarified cell extract using cobalt or nickel His-select affinity gel purification column (Sigma). Protein was eluted in 250mM imidazole and further purified using an Electro-Eluter (Bio-Rad, Hercules CA, USA). Dialyzed eluted fractions were concentrated with an Amicon Ultra 3.5 kD MWCO cutoff filter (MilliporeSigma, Burlington MA, USA). Protein purity was assessed by SDS-PAGE electrophoresis followed by staining with SimplyBlue SafeStain (Invitrogen). Purified protein was quantified by bicinchoninic acid analysis (Bio-Rad).

### Antibody production

One BALB/c mouse was immunized with 20 μg of recombinant Wb-Bhp-1 in complete Freund’s adjuvant and boosted 1 month later with 20 μg of Wb-Bhp-1 in incomplete Freund’s adjuvant. Immune serum was collected 9 days after the boost. Serum from an unimmunized BALB/c mouse serum was used as a negative control.

### Tissue fixation and immunohistochemistry

Immunochemistry was performed on fixed and sectioned *B*. *malayi* parasites recovered at various stages of development from intraperitoneally infected jirds, as well as on fixed and sectioned *O*. *volvulus* nodules, which were originally obtained from an onchocerciasis patient in Ghana [33,34]. The parasites were fixed in either 4% formaldehyde or in 80% ethanol then embedded in paraffin. Immunostaining was conducted with the alkaline phosphatase anti-alkaline phosphatase (APAAP) method as previously described [33]. Primary antibody dilutions of 1:100 to 1:1000 were assessed, and the dilutions 1:200 and 1:500 were found to provide the best signal over background. Polyclonal rabbit anti-mouse IgG 1:25 (Dako, Santa Clara CA, USA) was used as the secondary antibody, then mouse APAAP of at a dilution of 1:40 (Sigma) was applied. The chromatogen Fast Red (Sigma) was used as the substrate and the slides were counter stained with hematoxylin (Merck, Darmstadt Germany). Slides were examined on an Olympus -BX40 microscope and photographed on an Olympus DP70 microscope digital camera (Olympus, Tokyo Japan).

### Immunoblot analysis

Purified recombinant proteins were separated by SDS-PAGE and transferred to nitrocellulose membranes (Bio-Rad). Membranes were blocked with 5% milk, then were probed with either antisera to the Xpress epitope (Invitrogen) at 1:4000 dilution or with patient sera diluted at 1:100. Blots were then incubated with secondary antibody anti-mouse IgG-alkaline phosphatase (Promega, Madison WI, USA), or anti-human IgG4-pFc’- alkaline phosphatase (Southern Biotech, Birmingham AL, USA) at 1:4000. Antibody binding was visualized by incubating blots in SigmaFast BCIP/NBT alkaline phosphatase substrate (Sigma).

### Indirect ELISA

96 well vinyl round bottom plates were coated with 100ul of 0.5 ug/ml of Wb-Bhp-1 in 0.06M carbonate buffer pH 9.6, covered and incubated at 37°C overnight in a humidified box. Plates were washed twice in PBS-Tween (PBST), then blocked with PBST-5% heat inactivated fetal calf sera (FCS) at 37°C for 1 hour. Human sera diluted at 1:100 in PBST-5% FCS was then added and plates were incubated at 37°C for 2 hours. Plates were washed 5 times with PBST, then anti-human IgG4-pFc’-HRP (Southern Biotech) diluted at 1:4000 in PBST-5% FCS was added and plates were incubated at 37°C for 1 hr. Plates were washed in PBST 5 times before adding the substrate o-phenylenediamine dihydrochloride (Thermo Fisher Scientific, Waltham MA, USA). The colorimetric reaction was stopped with 4M H_2_SO_4_ and plates were read at 490 nm with a BioTek ELx808 plate reader (Thermo Fisher Scientific). A positivity cutoff of OD_490_ > 0.2 was chosen to maximize sensitivity and specificity. Data were analyzed in Excel (Microsoft, Redmond WA, USA) and Prism version 9 (GraphPad, San Diego CA, USA). Biotinylated Wb-Bhp-1 peptide fragments (synthesized by LifeTein, Somerset NJ, USA) were used for ELISA as described above, on a plate coated with 10ug/ml streptavidin (Sigma).

### Human samples

De-identified sera and plasma samples were from individuals infected with a single filarial parasite unless otherwise noted. We tested sera or plasma from patients infected with *W*. *bancrofti*, *B*. *malayi*, *O*. *volvulus* and *L*. *loa* (Table 1). De-identified non-endemic control sera were obtained from Barnes Jewish Hospital clinical lab in St. Louis, Missouri. Since the St. Louis population has low rates of travel or emigration from regions endemic for filarial infections, these samples were presumed to be from non-exposed individuals.

**Table 1:** Sera characteristics.

Infection	Location of sera collection	Number of sera	Filarial co-infections	Diagnosis method	Citation
*W*. *bancrofti*	India	26	Absent	Mf count by blood smear or filtered blood	[35]
	Sri Lanka	39	Absent	Mf count of filtered blood	[36]
	Egypt	28	Absent	Mf count of filtered blood	[37]
	Papua New Guinea	23	Absent	Mf count of filtered blood	[2,3]
	Côte d’Ivoire	108	*M*. *perstans* possible	Mf count of filtered blood	[38,39]
*B*. *malayi*	Kerala, India	12	Absent	Mf count by blood smear or filtered blood	[35,40]
*O*. *volvulus*	Uganda	12	*M*. *perstans* possible	Mf count of skin snip	[41]
	Cameroon	10	*M*. *perstans* possible	Mf count of skin snip	[42]
	Nigeria	10	*M*. *perstans* possible	Mf count of skin snip	[43]
*L*. *loa*	Cameroon	29	59% *M*. *perstans*	Mf count by blood smear	[44]
Non-endemic control	St. Louis, USA	48	Absent^a^	Not applicable	

^a^ Non-endemic control serum from USA presumed free from filarial infection

### Ethics statement

Sera from patients infected with *W*. *bancrofti* were collected during the studies cited in Table 1. All clinical samples were de-identified and data regarding the samples and infections were labeled by study identification number only. De-identified non-endemic control sera samples were obtained de-identified from the Barnes-Jewish-Christian Hospital Clinical laboratory in St Louis. The Washington University in St Louis Human Research Protection Office (an institutional review board) determined that work with these de-identified samples did not constitute human subjects research.

### Statistical analysis

Statistical analysis was conducted in Prism Version 9 (GraphPad). Comparisons between antibody levels from groups of patients utilized Kruskal-Wallis one-way analysis of variance.

## Results

### Identification and production of BmR1 homologues

We identified two *W*. *bancrofti* homologues of *Bm17DIII*, the *B*. *malayi* gene that encodes BmR1. The first encodes a hypothetical protein (Genbank accession number EJW70263.1, UniProt ID J9DKX0). We named this gene *Wb-bhp-1* according to the recommended filarial nomenclature [45]. We amplified this gene from *W*. *bancrofti* genomic DNA and the gene was Sanger sequenced and submitted to Genbank (accession number OL692807). Interestingly, *Wb-bhp-1* has only one exon while *Bm17DIII* has two. We also identified another BmR1 homologue in *W*. *bancrofti*, termed *Wb-bhp-2*, which has two exons like *Bm17DIII*. The homologue from *O*. *volvulus*, OVOC7606.1, encodes a hypothetical protein (UniProt ID A0A2K6WDP0). We named this gene *Ov-bhp-1*. The best hit from *L*. *loa* was EN70_10598 and encodes a hypothetical protein (UniProt ID A0A1I7V734_LOALO). We named this gene *Ll-bhp-1*. Both *Ov-bhp-1* and *Ll-bhp-1* contain 2 exons.

**Fig 1:**
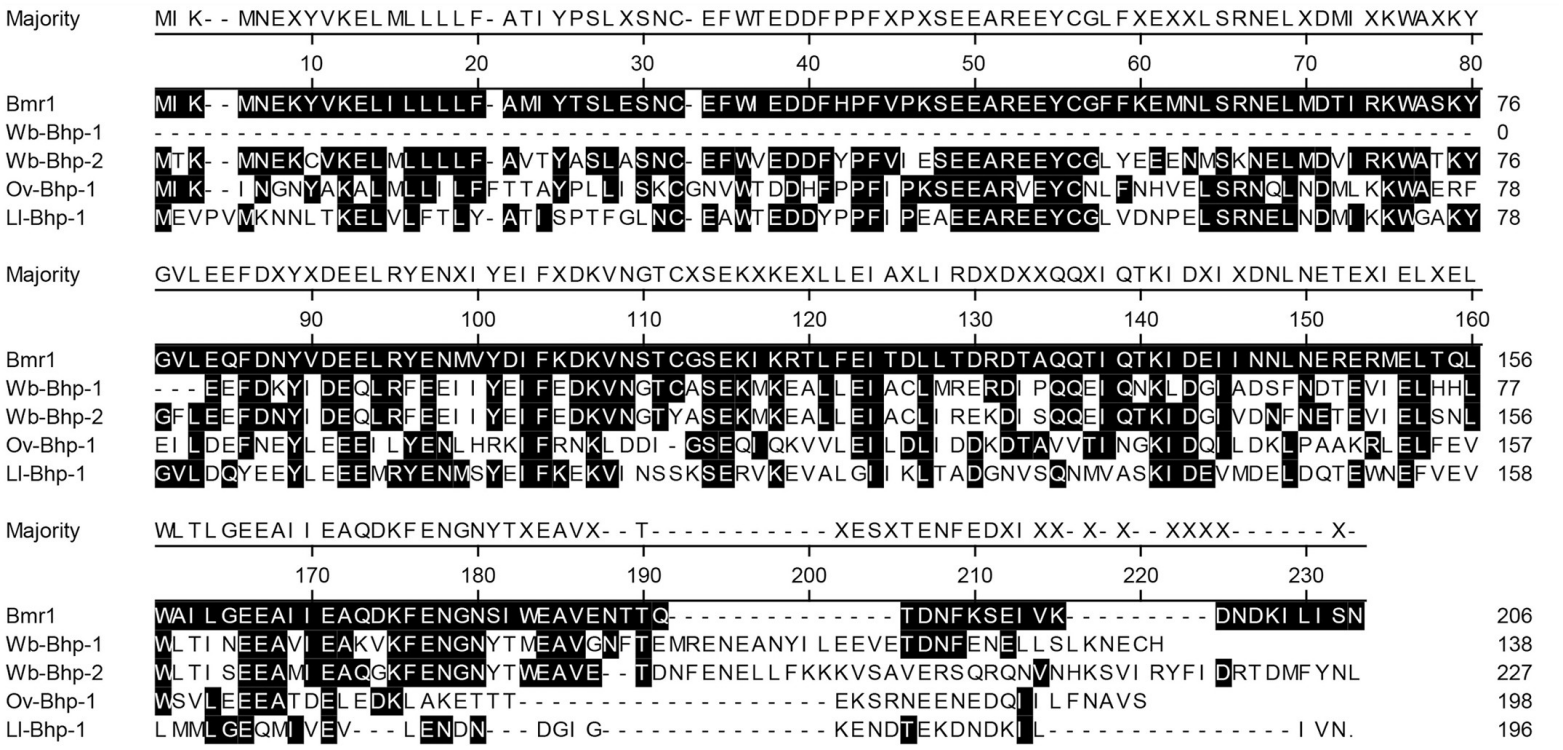
Alignment of BmR1 homologous proteins. Amino acid alignment by MegAlign and percent identity to *B*. *malayi* BmR1 of the following BmR1 homologous proteins: *W*. *bancrofti* Wb-Bhp-1 (45%) and Wb-Bhp-2 (55%), *O*. *volvulus* Ov-Bhp-1 (41%), and *L*. *loa* Ll-Bhp-1 (42%). Amino acid residues conserved with BmR1 are shaded black.

Because the functions of BmR1 and these newly identified homologues are unknown, we named these homologues BmR1 homologous protein (Bhp), with the homologue in each species identified by the standard species acronym [45]. Wb-Bhp-1 has 45% amino acid identity to the portion of BmR1 encoded by the second exon. Ov-Bhp-1 and Ll-Bhp-1 have 41% and 42% amino acid identity to BmR1, respectively. The amino acid alignments of these newly identified homologues to BmR1 are shown (Fig 1). They are unlike previously identified BmR1 homologues which had 99–100% amino acid identity to BmR1 [24]. BmR1 also has homologues in more distantly related nematodes including *Dirofilaria immitis* and *Toxocara canis*. However, those homologues demonstrate only 22–33% amino acid identity to BmR1 and 22–28% identity to Wb-Bhp-1 [46,47].

**Fig 2:**
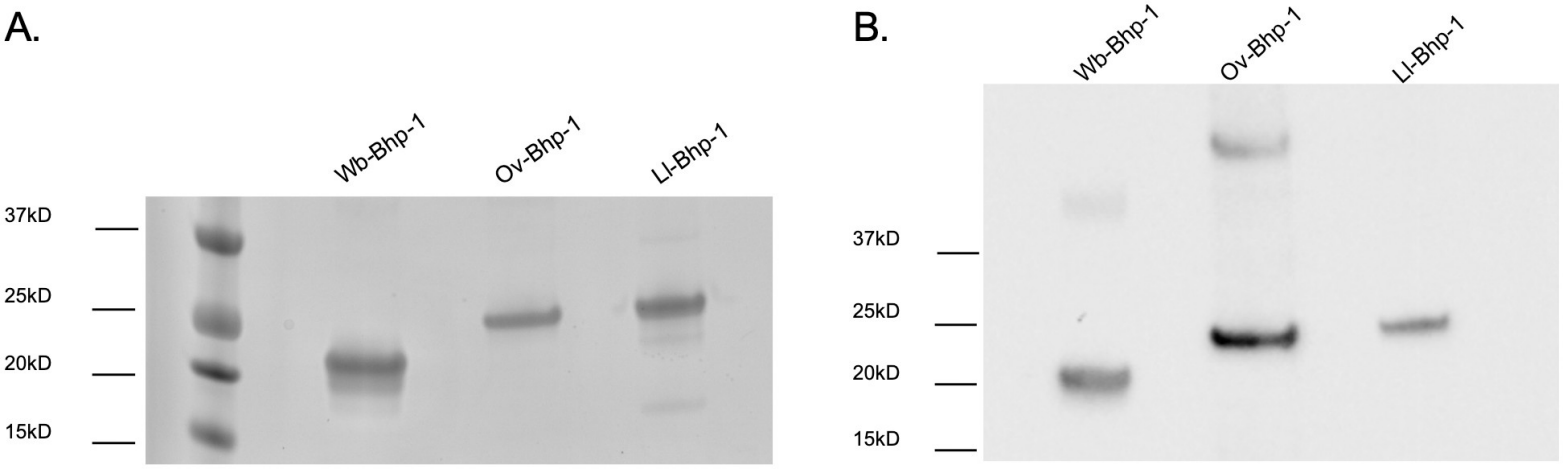
Characterization of BmR1 homologous proteins. (A) SDS-PAGE and SimplyBlue SafeStain analysis of recombinant Wb-Bhp-1 (predicted 20.4 kD), Ov-Bhp-1 (predicted 27 kD), and Ll-Bhp-1 (predicted 26.5 kD). (B) Immunoblot analysis of Wb-Bhp-1, Ov-Bhp-1, Ll-Bhp-1 with anti-Xpress antibody, which targets the amino-terminal Xpress epitope of these recombinant proteins.

In order to evaluate Wb-Bhp-1 as a diagnostic candidate, we cloned *Wb-bhp-1* into an expression vector that incorporates an amino-terminal His tag and Xpress epitope to allow for protein localization. This recombinant protein has a predicted molecular weight of 20.4 kilodaltons (kD), and was purified utilizing the N-terminal His tag. The protein was visualized at the expected molecular weight by SDS-PAGE and immunoblot analysis with anti-Xpress antibody (Fig 2A and 2B). A dimer of the recombinant protein is also visible (Fig 2B). Wb-Bhp-2 was also characterized but is not presented here because of its lower assay specificity relative to Wb-Bhp-1, as discussed below.

### Immunohistochemical localization of BmR1 homologues

**Fig 3:**
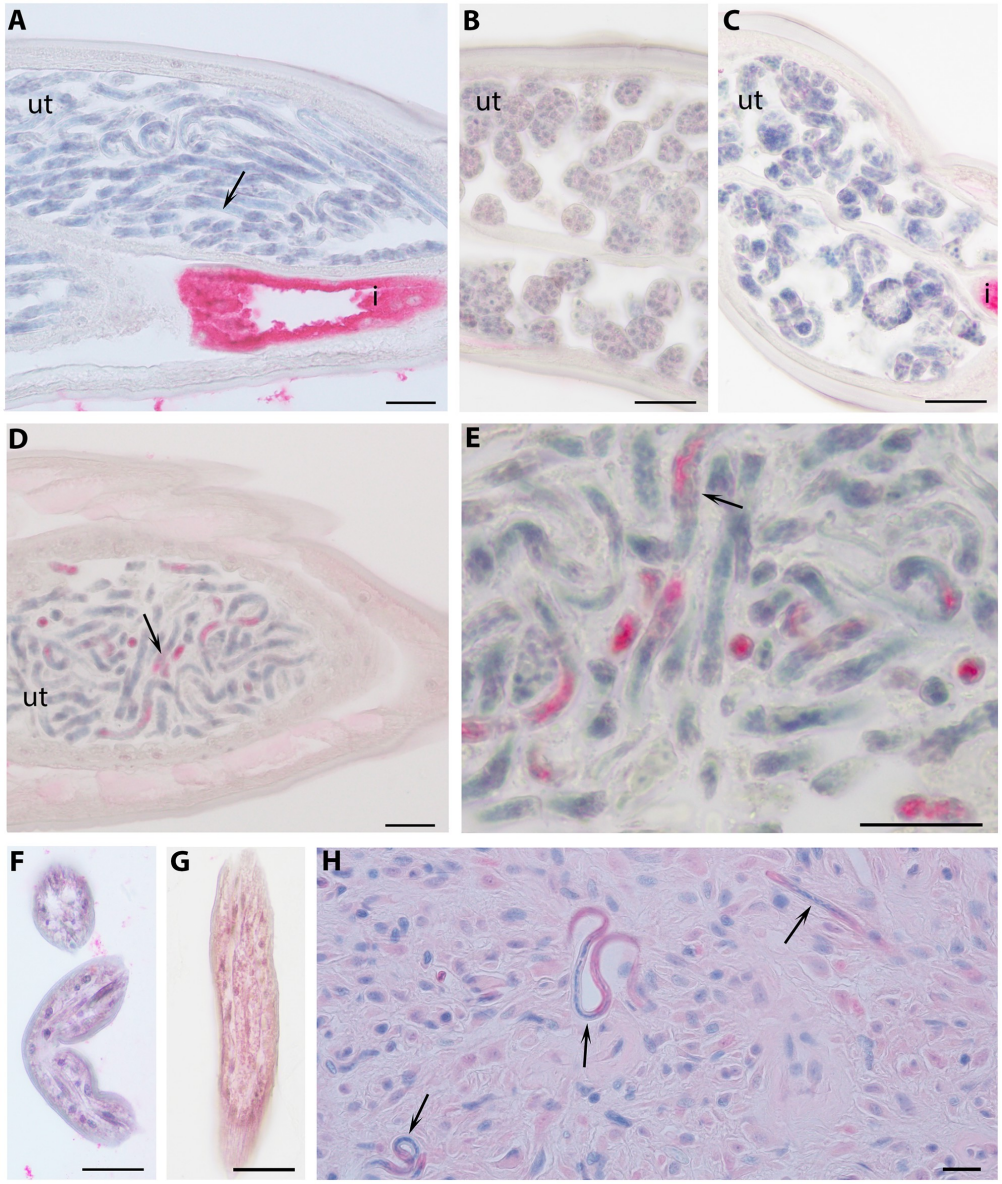
Immunolocalization of BmR1 homologues. This figure shows immunohistochemical results from worm sections that were processed as described in the Methods. A *B*. *malayi* female worm section stained with normal mouse sera showed no labeling (A). *B*. *malayi* female worm sections stained with anti-Wb-Bhp-1 demonstrated no staining in morula (B) or pretzel stage larvae (C) but staining of stretched Mf (DE). *B*. *malayi* L3 stage demonstrated minimal staining with normal mouse sera (F) and minimal staining in a diffuse pattern with anti-Wb-Bhp-1 sera (G). *O*. *volvulus* nodule stained with anti-Wb-Bhp-1 demonstrated staining of Mf within the nodule (H). Annotations: Mf (arrow), uterus (ut), and intestine (i), which has intrinsic alkaline phosphatase. Scale bar indicates 20 micrometers.

Unlike *B*. *malayi Bm17DIII*, nothing was known about expression profile of *Wb-bhp-1*, or the localization of Wb-Bhp-1 in filarial worms. We raised polyclonal mouse antibodies to Wb-Bhp-1 in order to further characterize this protein. Because *W*. *bancrofti* adult worms and Mf are challenging to obtain, we utilized *B*. *malayi* parasites for localization studies. Mouse anti-Wb-Bhp-1 antibodies bound to the stretched Mf in adult female worms of *B*. *malayi* (Fig 3D and 3E). There was no staining of *B*. *malayi* oocytes, morula, or pretzel stage Mf (Fig 3B and 3C). There was slight diffuse staining of *B*. *malayi* L3 stage parasites that we believe represents background staining (Fig 3G). There was no staining of *B*. *malayi* Mf by control mouse serum (Fig 3A). There was no staining in male worms. Mouse antibodies to recombinant Wb-Bhp-1 also bound to Mf in *O*. *volvulus* nodules, especially those free in the nodule rather than in utero (Fig 3H). These results suggest that antibodies to recombinant Wb-Bhp-1 cross reacted with *B*. *malayi* and *O*. *volvulus* homologous proteins.

### Antibodies to Wb-Bhp-1 detectible in sera from people with *W*. *bancrofti* microfilaremia.

**Fig 4:**
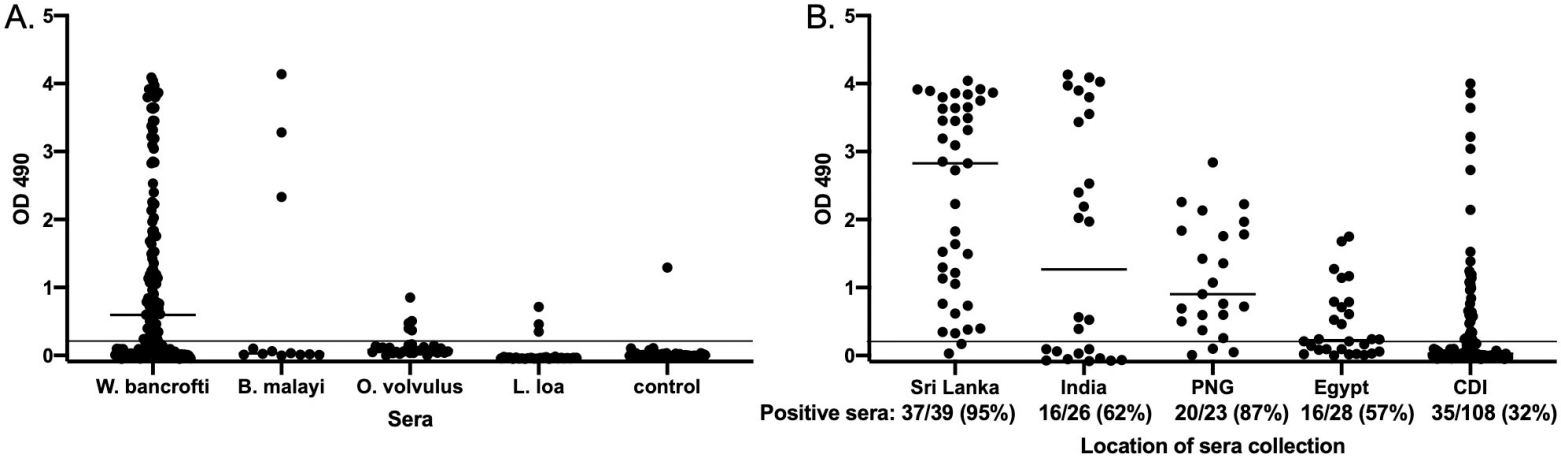
Anti-Wb-Bhp-1 IgG4 ELISA sensitivity and specificity. Graphs show the individual OD_490_ for the anti-Wb-Bhp-1 IgG4 ELISA. Median values are indicated by the black bar. Positivity cutoff of 0.2 is indicated by the dotted black line. PNG: Papua New Guinea. CDI: Côte d’Ivoire. (A) ELISA data for sera from people with the indicated filarial infection, or non-endemic controls (Table 1). Median ELISA data from each filarial infection are statistically different by Kruskal-Wallis test with p< 0.0001. (B) ELISA data from individuals with *W*. *bancrofti* infection from the specified country of origin. Median OD_490_ and overall percent positivity for each country are significantly different by Kruskal-Wallis, with p<0.0001.

We screened sera from people infected with *W*. *bancrofti*, and 12 of 13 (92%) reacted with Wb-Bhp-1 by immunoblot. We then evaluated patient sera by ELISA. IgG4 subclass was used in the ELISA as this IgG subclass is usually the most specific for anti-filarial diagnostics [13,23]. We also developed and evaluated an anti-IgG ELISA, but it was less specific than the IgG4 ELISA. We tested 224 sera from individuals with *W*. *bancrofti* microfilaremia collected in Sri Lanka, India, Papua New Guinea, Egypt and Côte d’Ivoire (Table 1). One hundred and twenty four of 224 (55%) sera had anti-Wb-Bhp-1 IgG4 ELISA titers above the threshold of 0.2 OD_490_ (Fig 4A). Median antibody levels and proportion of individuals with anti-Wb-Bhp-1 IgG4 antibodies varied significantly between samples from different countries (Fig 4B). Samples from Sri Lanka demonstrated a median OD_490_ value of 2.7, as well as 95% sensitivity, the highest from any location, while samples from Côte d’Ivoire had the lowest median OD_490_ at 0.03, and the lowest sensitivity at 32%. Because the assay had low sensitivity with the initial 56 samples from Côte d’Ivoire, we tested an additional 51 sera collected from a different cohort of Mf positive individuals in Côte d’Ivoire. The second set of samples had similar median OD_490_ and percent positivity to the first sera set. Fig 4 shows results for all the samples from Côte d’Ivoire.

**Fig 5:**
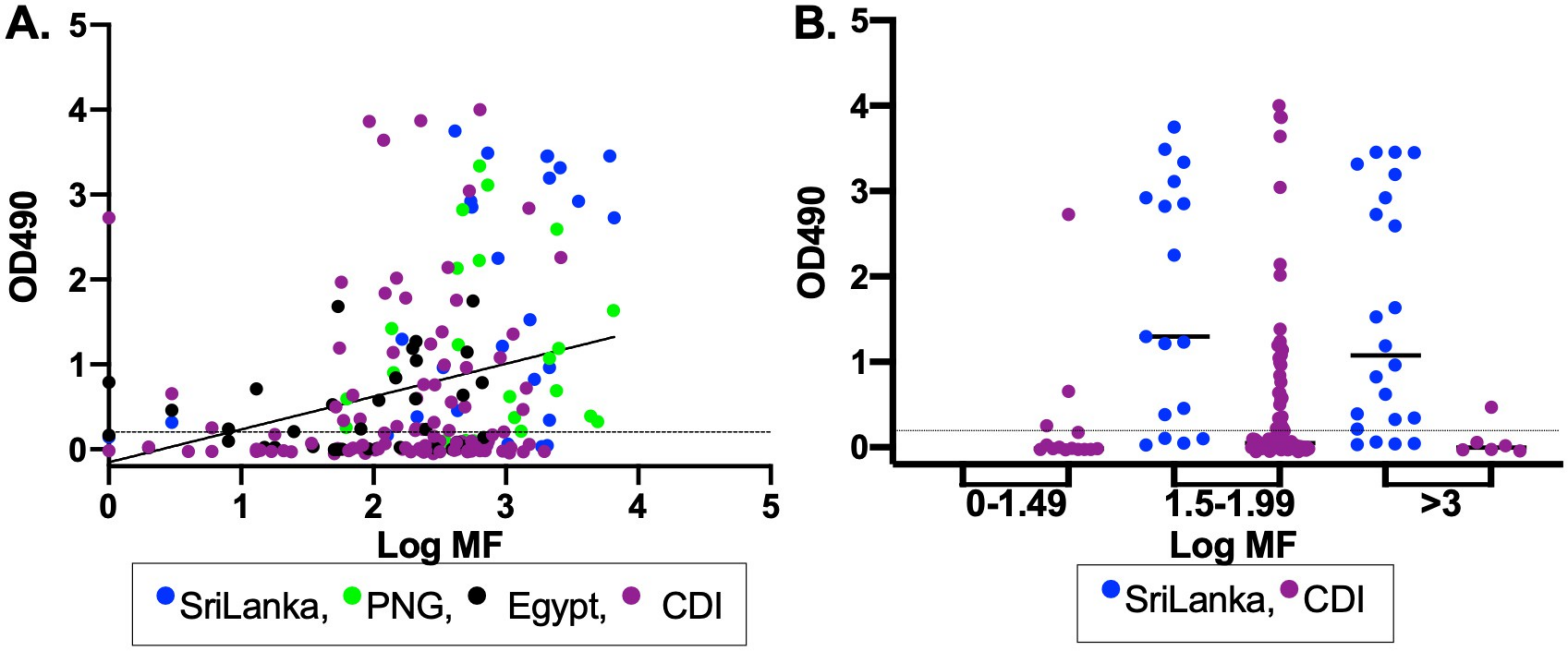
Anti-Wb-Bhp-1 IgG4 ELISA dependence on Mf count. Graphs show the individual OD_490_ for the anti-Wb-Bhp-1 IgG4 ELISA. Median values are indicated by black bar. Positivity cutoff of 0.2 is indicated by the dotted black line. Data are color coded by country of origin for the sera. PNG: Papua New Guinea. CDI: Côte d’Ivoire. (A) OD_490_ titers plotted against the log of Mf count. Values are significantly correlated by Spearman, with r = 0.2982, p<0.0001. (B) OD_490_ titers from individuals with *W*. *bancrofti* infection from the specified country of origin stratified by log of Mf count. Median OD_490_ and overall percent positivity for each column are significantly different by Kruskal-Wallis, with p< 0.0001.

Anti-IgG4 ELISA OD values were positively correlated with Mf count (Fig 5A). This analysis included 198 samples where Mf counts were performed by membrane filtration. This analysis does not include samples from India where Mf counts were performed with blood smears that do not provide comparable counts to membrane filtration. The sensitivities of anti-Wb-Bhp-1 IgG4 ELISA for samples from people with Mf counts of 1 to 30 (Log Mf of 0–1.49), 31 to 999 (Log Mf of 1.5–2.99), and ≥ 1000 per ml of blood (Log Mf of ≥ 3) were 35%, 50%, and 74%, respectively. Sample sets from all locations included plasma from people with a range of Mf counts. However, 56% of the Sri Lanka samples were from people with Mf counts ≥ 1000 per ml, while only 6% of samples from Côte d’Ivoire had Mf counts in that range. This difference in Mf count for samples from various locations contributed to the wide variation in median OD_490_ and sensitivity that we observed in ELISA results for samples from different countries, but does not explain it completely. There were significantly different median values and sensitivities of the anti-Wb-Bhp-1 IgG4 ELISA in samples from people from Sri Lanka and Côte d’Ivoire even after stratifying by Mf count (Fig 5B). All samples from different regions were collected from microfilaremic patients who had no recent history of treatment for lymphatic filariasis.

We investigated whether there was sequence variability in the *Wb-bhp-1* gene from *W*. *bancrofti* isolated in different endemic areas. A recent study on *W*. *bancrofti* genomes utilized 47 single worms isolated from Haiti, Mali, Kenya and Papua New Guinea [25]. Our analysis showed that there is sequence variability within various *Wb-bhp-1*, with 11 single nucleotide polymorphisms compared to the sequence of *Wb-bhp-1* sequenced for this work, 9 of which are missense mutations (S1 Fig). One of these polymorphisms is within a region predicted to be a surface epitope on BmR1 [48].

IDA is the most effective therapeutic regimen known for clearing *W*. *bancrofti* Mf, but it cannot be used in *O*. *volvulus* endemic regions because it contains DEC. The anti-Wb-Bhp-1 IgG4 ELISA might be especially useful for monitoring LF elimination from a community after MDA with IDA. Therefore, we calculated the sensitivity of the anti-Wb-Bhp-1 IgG4 ELISA using sera from countries where IDA can be used for MDA (this excluded the samples from Côte d’Ivoire which is endemic for *O*. *volvulus*). The sensitivity of the anti-Wb-Bhp-1 IgG4 ELISA was 77% for the 116 samples from IDA eligible countries (Sri Lanka, India, Papua New Guinea and Egypt).

### Specificity of the Wb-Bhp-1 IgG4 ELISA

Anti-Wb-Bhp-1 antibody levels in sera from people infected with *O*. *volvulus* and *L*. *loa* are shown **(**Fig 4A). Five of 30 (17%) onchocerciasis samples and 3 of 29 (10%) of loiasis samples had IgG4 antibodies to Wb-Bhp-1 by ELISA. Thus, the Wb-Bhp-1 ELISA has an estimated specificity of 83% for samples from Mf-positive people with onchocerciasis and 90% for people with loiasis. We also tested samples from people with *B*. *malayi* microfilaremia for anti-Wb-Bhp-1 antibodies. *W*. *bancrofti* and *B*. *malayi* are closely related parasites, and the Brugia Rapid test has 50–70% sensitivity for *W*. *bancrofti* infection, depending on the format of the diagnostic used [49,50]. However, only 3 of 12 (25%) samples from people with *B*. *malayi* infection had antibodies reactive to Wb-Bhp-1 (Fig 4A).

We also identified another BmR1 homologue in *W*. *bancrofti*, Wb-Bhp-2. This antigen is less promising as a diagnostic because of lower specificity compared to Wb-Bhp-1, with 20% of onchocerciasis, 10% of loiasis patient sera and 4.3% of control sera containing cross reactive antibodies to Wb-Bhp-2. It is possible that the increased cross reactivity of Wb-Bhp-2 is due to surface epitopes encoded by the first exon of *Wb-bhp-2*.

In silico structural analysis of BmR1 identified three putative surface exposed epitopes [48]. We examined whether the corresponding regions on Wb-Bhp-1 might be epitopes that could provide increased specificity in a peptide ELISA. We tested biotinylated peptides of the 2 predicted surface epitopes within Wb-Bhp-1 with the amino acid sequences RERDIPQQEIQNKLDGIADSFNDT and VNGTCASEK. Unfortunately, IgG4 ELISA using these biotinylated peptides demonstrated that 6 of 9 (67%) people with onchocerciasis and 2 of 5 (40%) of people with loiasis also contained antibodies to these peptides.

Because *Mansonella perstans* is co-endemic with other filarial infections in Africa, it is possible that it may contribute to cross-reactivity. Some of the sera used in the study were obtained from people with onchocerciasis and loiasis who were also infected with *M*. *perstans*, (Table 1). However, only 1 of 14 (7%) samples from people co-infected with loiasis and *M*. *perstans* had antibodies to Wb-Bhp-1, suggesting that the rate of cross-reactivity among co-infected individuals is not higher than among those with *L*. *loa* alone.

We identified antibodies reactive to Wb-Bhp-1 by IgG4 ELISA in 2 of 60 samples from St. Louis (USA), a non-endemic region for these filarial infections in humans (Fig 4A). We cannot exclude the possibility that these deidentified samples were from immigrants or travelers with prior exposure to filarial infections. However, if we assume that none of these serum donors had such exposures, the specificity of the anti-Wb-Bhp-1 IgG4 ELISA was 96.7% with non-endemic control serum samples.

### Antibodies to Ov-Bhp-1 and Ll-Bhp-1

**Fig 6:**
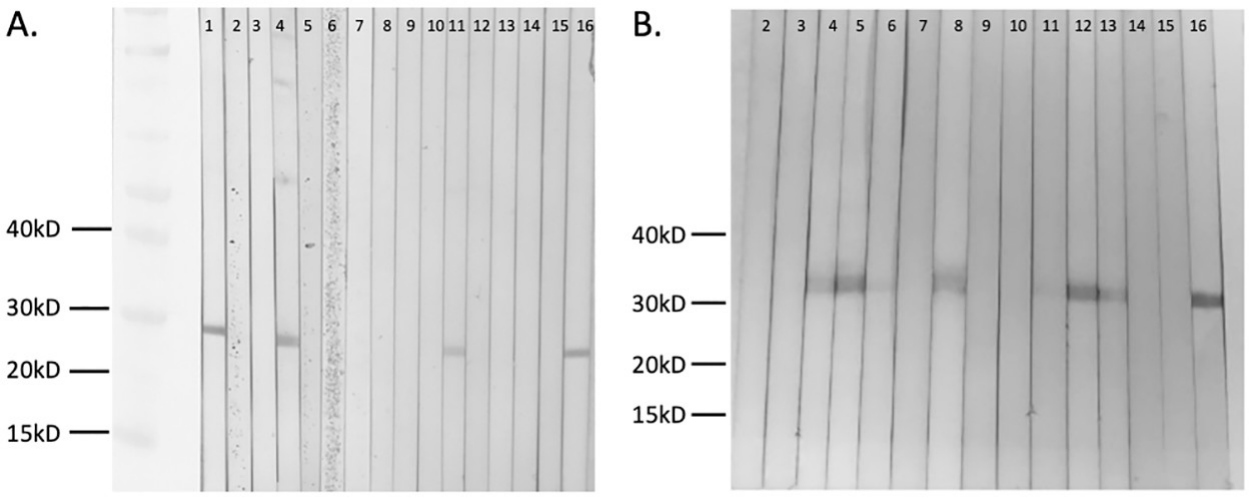
Identification of human antibodies to Ov-Bhp-1 and Ll-Bhp-1. (A) Representative immunoblot results for Ov-Bhp-1with sera from people with onchocerciasis. (B) Representative immunoblot analysis of Ll-Bhp-1 with sera from people with loiasis. Lanes 1 and 16 used anti-Xpress control antibody. Lanes 2–15 show immunoblot results with sera from people with the specified infection.

As described above, we also cloned and expressed *Ov-bhp-1* and *Ll-bhp-1* to evaluate their potential as diagnostic candidates for onchocerciasis and loiasis. The soluble proteins Ov-Bhp-1 and Ll-Bhp-1 had the expected molecular weights of 27 and 26.5 kD, respectively, based on SDS-PAGE and immunoblot analysis with anti-Xpress antibody (Fig 2A and 2B). We then performed immunoblot analyses with sera from patients with onchocerciasis and loiasis (Table 1), and representative immunoblots are shown (Fig 6A and 6B). Only 3 of 17 (17.6%) onchocerciasis samples and 10 of 29 (34%) of loiasis samples contained antibodies to Ov-Bhp-1 or Ll-Bhp-1, respectively. These results suggest that the BmR1 homologues Ov-Bhp-1 and Ll-Bhp-1 are not useful diagnostic antigens for onchocerciasis or loiasis.

## Discussion

Antibody-based diagnostic tests could be quite useful as assessment tools for LF elimination programs. They can be formatted into inexpensive lateral flow assays that are especially useful in low resource settings. Antibodies to some filarial antigens could be more sensitive than CFA tests or Mf for demonstrating infection or exposure in surveys of school children [9,11]. However, children are sometimes not valid sentinels for this purpose in areas where filarial infections are much more common in adults than in children. Antibody tests that correlate well with the presence of microfilaremia would be quite useful for assessing the risk of ongoing transmission in areas that have received several rounds of MDA. This is especially true for areas that have received MDA with the triple drug regimen IDA, because CFA tends to persist long after Mf have been cleared by IDA. Assays like the Brugia Rapid test that reverts to negative relatively quickly after effective treatment might be especially useful for assessing the risk of ongoing transmission after MDA with IDA [16]. In this study, we identified, cloned, purified and characterized Wb-Bhp-1, a BmR1 homologue from *W*. *bancrofti*. Immunolocalization studies showed that anti-Wb-Bhp-1 antibodies bind to protein in *B*. *malayi* Mf. This result was consistent with the expression patterns reported for *Bm17DIII* and BmR1 [17,18,51–53]. Anti-Wb-Bhp-1 antibodies bound to a target in *O*. *volvulus* Mf, reinforcing the link between this antibody and filarial Mf.

The variable sensitivity of the Wb-Bhp-1 ELISA with *W*. *bancrofti* sera from different countries was unexpected, and it is only partially explained by the relationship between ELISA results and Mf density. More work is needed to explain why the ELISA was less sensitive for *W*. *bancrofti* samples from Côte d’Ivoire, and samples from other areas in sub-Saharan Africa need to be tested to verify whether this low sensitivity is a problem in other parts of Africa. Geographic variability in *Wb-bhp-1* might explain this finding. However, our analysis of *Wb-bhp-1* sequences in parasites collected in diverse locations did not reveal enough sequence variation to explain the variability in antibody responses that we observed. Côte d’Ivoire is the only country with samples in this study where LF is co-endemic with onchocerciasis. However, it is unclear why coinfection with onchocerciasis would reduce antibody responses to Wb-Bhp-1.

On the positive side, the Wb-Bhp-1 ELISA had a sensitivity of 77% for samples from microfilaremic individuals in Sri Lanka, India, Papua New Guinea and Egypt, which are all areas that do not have co-endemic onchocerciasis. Therefore, Wb-Bhp-1 serology may be especially useful in countries where IDA can be used to accelerate LF elimination. Additional studies will be needed to assess the value of this test for population-based sero-surveys with samples from children and adults before and after MDA.

Specificity testing showed that the Wb-Bhp-1 ELISA had low-level cross-reactivity with samples from people with onchocerciasis or loiasis. This is essential for an assay to be useful for LF serology in many areas in sub-Saharan Africa. The relatively low sequence identity of homologues in *O*. *volvulus* and *L*. *loa* likely contributes to the specificity of the Wb-Bhp-1 ELISA. Interestingly, few sera from people with onchocerciasis or loiasis contained antibodies to the homologous proteins Ov-Bhp-1 or Ll-Bhp-1. It is possible that there are differences in localization, or expression of these proteins in those parasites that impact host antibody responses.

In conclusion, these studies have shown that Wb-Bhp-1, a *W*. *bancrofti* homologue of BmR1, is a promising microfilarial antigen for diagnosis of bancroftian filariasis. Additional studies are needed to characterize the effect of treatment and MDA on antibody levels in individuals and on antibody prevalence in populations. Results of those studies will determine the practical value of Wb-Bhp-1 antibody testing as a surveillance tool for LF elimination programs.

## Supporting information

S1 Table Strains produced and primers used for cloning. (DOCX)

S1 Fig Single nucleotide polymorphisms in *Wb-bhp-1*. (A) Graph demonstrates the 9 missense polymorphisms identified in Wb-Bhp-1. The amino acid with the mutation is plotted against the frequency with which each mutation is identified in *W*. *bancrofti* worms from the indicated country. The shaded regions represent 2 of the 3 putative antigenic surface epitopes identified in BmR1 (48). (B) Table indicates the specific polymorphisms identified relative to *Wb-bhp-1* sequence, as well as the frequency with which each polymorphism is identified in each country.
(TIF)
