# Supplementary material for: Characterization of a novel microfilarial antigen for diagnosis of Wuchereria bancrofti infections
Source: PLoS Negl Trop Dis. 2022 May 23;16(5):e0010407. doi: 10.1371/journal.pntd.0010407 (PMC9126377; doi:10.1371/journal.pntd.0010407)
Supplement: S1 Table — (DOCX) [file pntd.0010407.s001.docx]

**S1 Table: Strains produced and primers used for cloning.**

| **Strain name** | **Strain Description** | **Primers used for DNA amplification (Forward, Reverse)** |
| --- | --- | --- |
| SEG1-34-7 | BL21 pET100: *Wb-bhp-1* | CACCATGTTAGAAAATACTAATGTACTACAGGAAGAATTTGAC TCAATGGCATTCATTTTTCAATGATAGCAGTTC |
| SEG1-34-1 | BL21 pET100: *Ov-bhp-1* | CACCATGATAAAAATCAATGGGAATTATGCTAAAGCATTG TTAAGAAACTGCATTAAATAAAATGATTTGATCTTCATTTTCTTC |
| SEG1-78-4 | BL21 pET100: *Ll-bhp-1* | CACCATGGAAGTGCCAGTAATGAAGAATAATTTGACC TTAATTTACGATCAATATCTTATCGTTGTC |
