# Supplementary figures and images for: Characterization of a novel microfilarial antigen for diagnosis of Wuchereria bancrofti infections
Source: PLoS Negl Trop Dis. 2022 May 23;16(5):e0010407. doi: 10.1371/journal.pntd.0010407 (PMC9126377; doi:10.1371/journal.pntd.0010407)

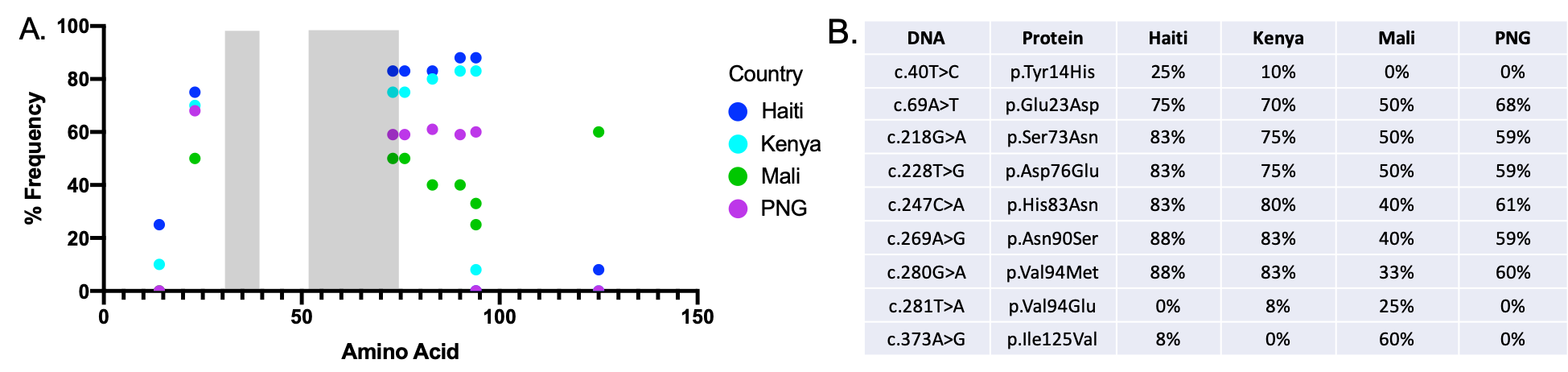

Supplement: S1 Fig — (A) Graph demonstrates the 9 missense polymorphisms identified in Wb-Bhp-1. The amino acid with the mutation is plotted against the frequency with which each mutation is identified in W. bancrofti worms from the indicated country. The shaded regions represent 2 of the 3 putative antigenic surface epitopes identified in BmR1 (48). (B) Table indicates the specific polymorphisms identified relative to Wb-bhp-1 sequence, as well as the frequency with which each polymorphism is identified in each country. (TIF) [file pntd.0010407.s002.tif]
